# A systematic review of student agency in international higher education

**DOI:** 10.1007/s10734-022-00952-3

**Published:** 2022-11-19

**Authors:** Kelsey Inouye, Soyoung Lee, Yusuf Ikbal Oldac

**Affiliations:** 1 Department of Education, University of Oxford, 15 Norham Gardens, Oxford, OX2 6PY UK; 2 School of Graduate Studies, Lingnan University, 8 Castle Peak Road, B.Y. Lam Building, LBY112, Lingnan, Tuen Mun, Hong Kong, People’s Republic of China

**Keywords:** International education, International students, Higher education, Agency, Systematic review, Educational sociology

## Abstract

The agency of international students has long been neglected and undertheorised, though recent literature indicates that this has started to change. This paper systematically reviews 51 studies that address student agency in international higher education. Focusing on research published in the last two decades (2000–2020), the review draws on studies that foreground student voices, or international students’ perspectives, rather than the perspectives of teachers, administrators or policymakers. A detailed discussion of how international student agency is positioned in the literature found that agency appears as either: a research object, as part of a theoretical or conceptual framework, or an emergent finding. Furthermore, our analysis suggests that the term “agency” is often used as a buzzword rather than as a fleshed-out concept. Thus, drawing on this initial analysis, the review synthesises varying but overlapping conceptualisations of international student agency in the literature into an integrative framework. Implications for future research are drawn, based on our findings about the understudied populations and methodological limitations in the literature.

**Supplementary Information:**

The online version contains supplementary material available at 10.1007/s10734-022-00952-3.

## Introduction 

### Context

Higher education has long had a cross-border element (Altbach, 1973), which can be traced back to mediaeval times (Kim, 2009). Recent decades have demonstrated an unprecedented increase in international activities in higher education, leading to a rise in policy discussions and research interests on different aspects of international higher education (IHE) (Altbach & Knight, 2007). Within IHE, international higher education students (“international students” henceforth) and their cross-border mobility have attracted the greatest attention, emerging as a prioritised topic (Knight, 2012) alongside the exponential increase in international student numbers over the last few decades. According to the UNESCO (2022), there were more than six million international students in 2019, up from approximately two million in 2000. This is a 300% increase in just two decades. Although this rising trend in international student mobility may have been curbed by the current COVID-19 pandemic and health-related uncertainties (Mok et al., 2021), it is expected to revert as the pandemic wanes (Altbach & de Wit, 2020).

Given the relevance of the international student population in higher education policy and development, an understanding of student experiences is important in furthering higher education research. We recognise that both structural forces and student agency are important and shape international student experiences (Oldac, 2022); however, the role of student agency remains under-explored (Tran & Vu, 2018; Volet & Jones, 2012). For instance, a recent review of the research on international students argued that dominant understandings of international student experiences are formed by “narratives of deficiency”, meaning that students tend to be framed in terms of “what they lack, what they need and how they differ”, contributing to the decades-long tendency to undermine international students’ agentic capacity (Lipura & Collins, 2020, p. 349). Recently, however, international higher education has been regarded as an important context to study student agency, as it provides a distinctive time and place where students actively deal with multi-level changes (regional, sociocultural and academic) away from home, maybe for the first time in their lives. We start our inquiry from an assumption that international students would experience these changes to a greater extent and in more aspects, which is shared by the dominant deficit discourse around international student experiences (e.g. Hechanova-Alampay et al, 2002).

The call for greater attention to agency in IHE is not new (see Brooks &Waters, 2011; Volet & Jones, 2012). Marginson (2014), for instance, has long highlighted the value of agency in bringing attention to “different observations and findings to those derived when cross-border students are positioned in a stress and coping framework” (p. 18). Yet, we, as researchers in higher education focusing on student agency, have noticed that despite growing interest in international students, agency remains relatively lacking in the literature (see also Lipura & Collins, 2020). Instead, “agency” sometimes appears as a buzzword, an idea that appears in research findings, discussions or literature reviews, but is not conceptually well-defined. This suggests that the concept of agency is still in the embryonic stage and requires further theorisation within the international student context. Our observations in combination with the observations of others in the field thus gave rise to the aims of this paper: (1) to review the existing literature on agency in IHE and the early development of the field, and (2) to synthesise how agency has been conceptualised by researchers. Additionally, we draw on the findings of this review to suggest directions for future research that may contribute to further conceptual development of student agency in IHE. In the following sections, we begin by providing an overview of the major existing theories of agency, and define the scope of our review, research questions and methodology. We then present a combined results and discussion section, organised in relation to each of our research questions.

### Theories of agency

In social theory, agency typically appears in relation to structure. Major social theories such as Archer’s (2003) morphogenesis theory, Giddens’s (1984) structuration theory and Bourdieu’s (1977) notion of habitus incorporate the agency-structure debate. While Archer interprets the structure-agency relation as independent, Giddens regards it as interdependent with structure internalised by agents (Akram, 2012). Located between Archer and Giddens, Bourdieu’s habitus views agents as autonomous and empowered, but only to the extent that structure is reproduced (Adams, 2006). To analyse what constitutes agency, Emirbayer and Mische (1998) define agency as constructing engagement with structure through agents’ reflection on the past, present and future. This idea influenced Biesta and Tedder’s (2007) conceptualisation of agency as one’s “ability to exert control over … one’s life” (p. 135) by means of structure rather than simply within structure. As such, agency is theorised in different ways based primarily on its relationship with structure.

In contrast, psychological theories of agency do not necessarily involve structure. For example, Bandura (2001) regards agency as a determinant of human behaviour, proposing four features of human agency: intentionality, forethought, self-regulation and self-reflectiveness. This implies that agency may be studied using a range of keywords. As we only include papers that use the term “agency”, this limits a more comprehensive discussion of agency as practiced in the world. 

### Scope of the review

We view agency as critical to understanding experiences of international students, highlighting the importance of individual trajectories and how structure—the national, cultural, institutional contexts etc.—facilitates or constrains international students’ ability to navigate and shape their educational experiences. In our own respective research, we have drawn on various conceptions of agency. However, because the aim of this review is to understand how agency has been positioned and conceptualised in the existing literature on IHE, we did not draw on a pre-existing theory of agency to guide our literature selection. Rather, we chose to include all relevant papers that reference “agency” in the abstract title or keywords. By focusing on the term “agency”, we can focus our discussion on both how agency has been understood and treated in the literature as well as on the phenomenon itself. However, as noted earlier, a focus on the word “agency” may preclude studies on psychological or other approaches to agency that uses different terminologies.

Our review defines “international students” as those who voluntarily move from their country of origin to another country (hereinafter “host country”) to pursue a higher education degree (OECD, 2017). In line with our focus on student agency, this systematic review focuses on the literature on international student voices, defined as research that draws upon the perspectives of international students themselves, rather than the perspectives of professors/lecturers, administrators or policymakers. Our focus derives from the call for greater examination of student experience to complement and inform policy-focused discussions on the internationalisation of higher education (Brooks & Waters, 2011).

We used the following questions to guide this study:

- Q1: How is agency positioned in the literature on student voices in international higher education?
- Q2: How can the literature on international student agency be conceptually synthesised?

These questions emerged from the inductive initial review of the selected papers as will be explained in the following sections.

## Methodology

### Literature search and selection

The literature search was conducted using SCOPUS, Web of Science, ProQuest Social Science, Eric and PsycINFO. We used a combination of keywords selected to capture studies focused on agency of international students at the undergraduate and postgraduate levels. See below for the search string.

(TITLE-ABS-KEY (agency OR agent*) AND TITLE-ABS-KEY (international OR global OR foreign OR mobil*) AND TITLE-ABS-KEY (“higher education” OR “tertiary education” OR college OR university) AND TITLE-ABS-KEY (student* OR undergraduate* OR post*graduate* OR graduate* OR doctora* OR phd* OR master*))

The search operating terms differed slightly for each database. Results were limited to peer-reviewed journal articles and book chapters published between 2000 and 2020 in English. English was chosen, as it is the lingua franca of the researchers. All database searches were conducted on February 8, 2021, and 5237 results were imported into Mendeley.

The titles were filtered and entries on irrelevant topics, for instance, medical studies, political studies and studies on secondary education, were deleted. Any editorials and commentaries were also deleted. A total of 4286 items were removed in the title filter process, resulting in 951 papers. Abstracts were read, and the inclusion/exclusion criteria (Table 1) were applied, resulting in 193 remaining items.

**Table 1:** Inclusion and exclusion criteria for abstracts

Criteria	Definition
Inclusion	
Focus on student voices	The focus of the paper must include student perspectives/experiences
Focus on degree mobile international students	The focus of the paper must be on degree mobile students, i.e. the student is pursuing an undergraduate or postgraduate degree in an institution not located in their country of origin; for joint degree programmes etc., the time spent abroad had to be one academic year minimum; shorter programmes and non-degree programmes (e.g. semester abroad, work/internship placement etc.) were excluded
Exclusion	
Instructor/institutional perspectives	Papers focusing only on perspectives of course instructors/lecturers/professors/supervisors, or institutional policy, were excluded
Online programmes	Papers focusing on online programmes were excluded; students must physically be studying in the host country
Curriculum	Papers focusing on developing or evaluating courses or programmes for international students were excluded

**Fig. 1:**
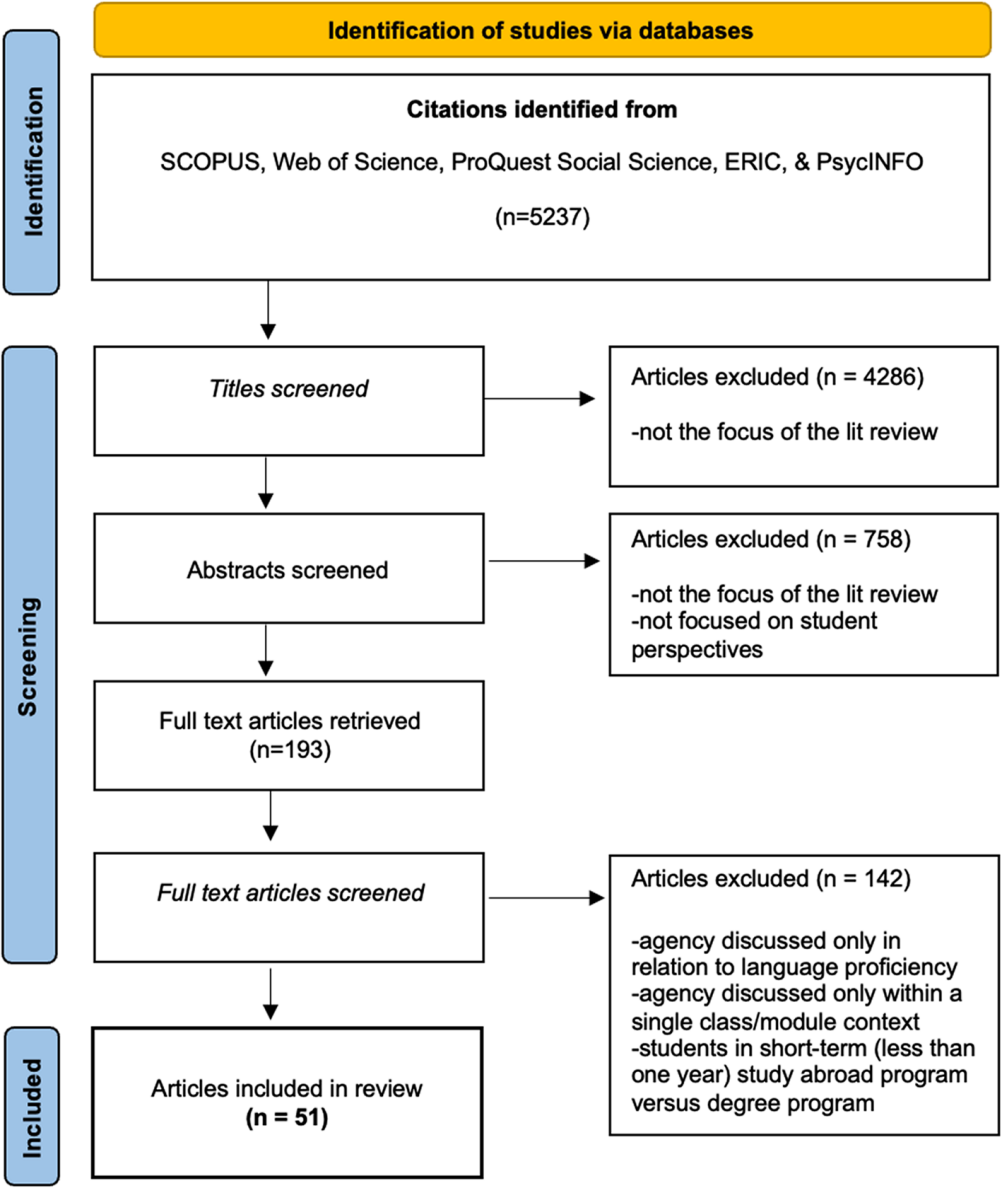
Flow chart of the article filtering process. Inspired from the PRISMA 2020 statement: an updated guideline for reporting systematic reviews. BMJ 2021;372;n71. https://doi.org/10.1136/bmj.n71

The full texts were retrieved and imported into Mendeley. Each paper was read through to confirm its relevance, using the criteria outlined in Table 2, which were developed jointly by the authors over several sessions of discussion. This process resulted in a final sample of 51 items (see Fig. 1 for the paper filtering process).

**Table 2:** Inclusion and exclusion criteria for full texts

Criteria	Definition
Inclusion	
Student agency	The paper must discuss international student agency in some capacity (e.g. in the research questions or in the results)
Exclusion	
Agency in language proficiency	Papers focusing on agency in using host country’s language in various settings were excluded
Agency within a single class/module	Papers focusing on students’ agency within the context of a single class or module (e.g. group work in classroom) were excluded
Agency prior to going abroad	Papers focusing on students’ agency prior to studying abroad (e.g. agency in choosing to pursue a degree abroad) were excluded
Agency of students from others’ perspectives	Papers in which others (e.g. instructors or institutions) discuss student agency were excluded
Transnational education	Papers on transnational education, e.g. students based in one country and studying for a degree at a university in another country, were excluded
Refugee/asylum seekers	Papers focusing on refugee or asylum seekers whose mobility is forced (i.e. not voluntarily pursuing a degree in another country) were excluded

### Analysis

The 51 included papers were read and categorised to determine how they positioned agency in their studies. We chose to examine the ways in which agency was manifested in the literature because in doing the full text filtering of the papers, we realised that agency appeared in a variety of places throughout the texts, sometimes in the research questions, other times in the theoretical framework to conceptualise international student decision-making and, often, in the results or discussion. We also noted that the term “agency” was not always well-defined or conceptualised and used to denote intention but framed as an assumed concept. We thus developed a framework to guide our coding, based on our observations from the full text reading (Table 3). This framework reflected the ways in which agency appeared/was positioned in the literature.

**Table 3:** Positioning of agency in the literature

Code/category	Definition
Agency as research object	Student agency is explicitly identified as the focus of the study or the research question(s)
Agency in conceptual/theoretical framework	Agency is defined by and integrated into the theoretical/conceptual framework
Agency as finding	Agency does not appear in the research focus, research questions or framework, but emerges as a finding
Agency as “given”	Agency is discussed within the paper, as research objects, frameworks or findings, but is not defined/conceptualised, and therefore is treated as having a “given” or assumed meaning
Agency in passing	Agency appears briefly within the paper, but is neither defined nor elaborated upon within any of the main sections above (research questions, framework, findings)

Upon developing our initial categories, we continued to refine the definitions over several iterations of coding and discussion. This process involved dividing the 51 included papers into thirds, and assigning two thirds of the corpus to each author to categorise independently as a way of ensuring that each paper was assessed by two authors. For instance, authors 1 and 3 worked on papers 1–17, authors 2 and 3 worked on papers 18–35, and authors 3 and 1 worked on papers 36–51. Papers could be double-coded if applicable. To illustrate, a paper could be categorised as both “agency as ‘given’” and “agency-as-finding” if agency appeared in the findings section but with no definition or conceptualisation. The results of the categorisation were then combined into a shared spreadsheet and discussed. Any discrepancies were examined and used to refine the initial categories and definitions, and another round of coding took place using the refined definitions. This process occurred several times until we were all in agreement. Subsequently, final categorisations were assigned for each paper.

### Limitations

Because our literature search was conducted in English, it is possible that we missed relevant work published in other languages. Likewise, papers not indexed in the databases we selected were not included. Our results may also be limited by our keywords. A keyword approach to the systematic review is useful in demonstrating patterns in the use of the terms, “agency” and “agent”, in research. However, the researched phenomenon can be examined under different headings. For instance, psychologists may discuss agency as self-efficacy, while Bourdieu talks about agency in various ways using different terms.^1^ Nevertheless, the keyword approach helped us to identify the growing popularity of choosing the term “agency” among other names, in discussing the phenomena that have multiple names. Furthermore, because the aim of this paper was to examine how the literature engages with the concept of agency, we believed it was important for agency to be explicitly named as a focus, rather than make interpretations about what could constitute agency under other terminologies. The consequence of this decision is that other potentially relevant papers examining phenomena that are linked to or an expression of agency (e.g. coping strategies, self-efficacy) may have been excluded.

## Findings and Discussion

The review followed an inductive approach guided by two research questions. This section begins with an overview of the trends identified in our analysis, followed by discussions of how the results address each of our research questions.

### Emerging insights: overarching trends in the literature

The 51 papers in our final sample were primarily qualitative (*n* = 46, 90.2%), while five papers used mixed methods (9.8%). None of the papers included in our sample used purely quantitative research designs, suggesting that agency is not easily studied through quantitative methods.

**Fig. 2:**
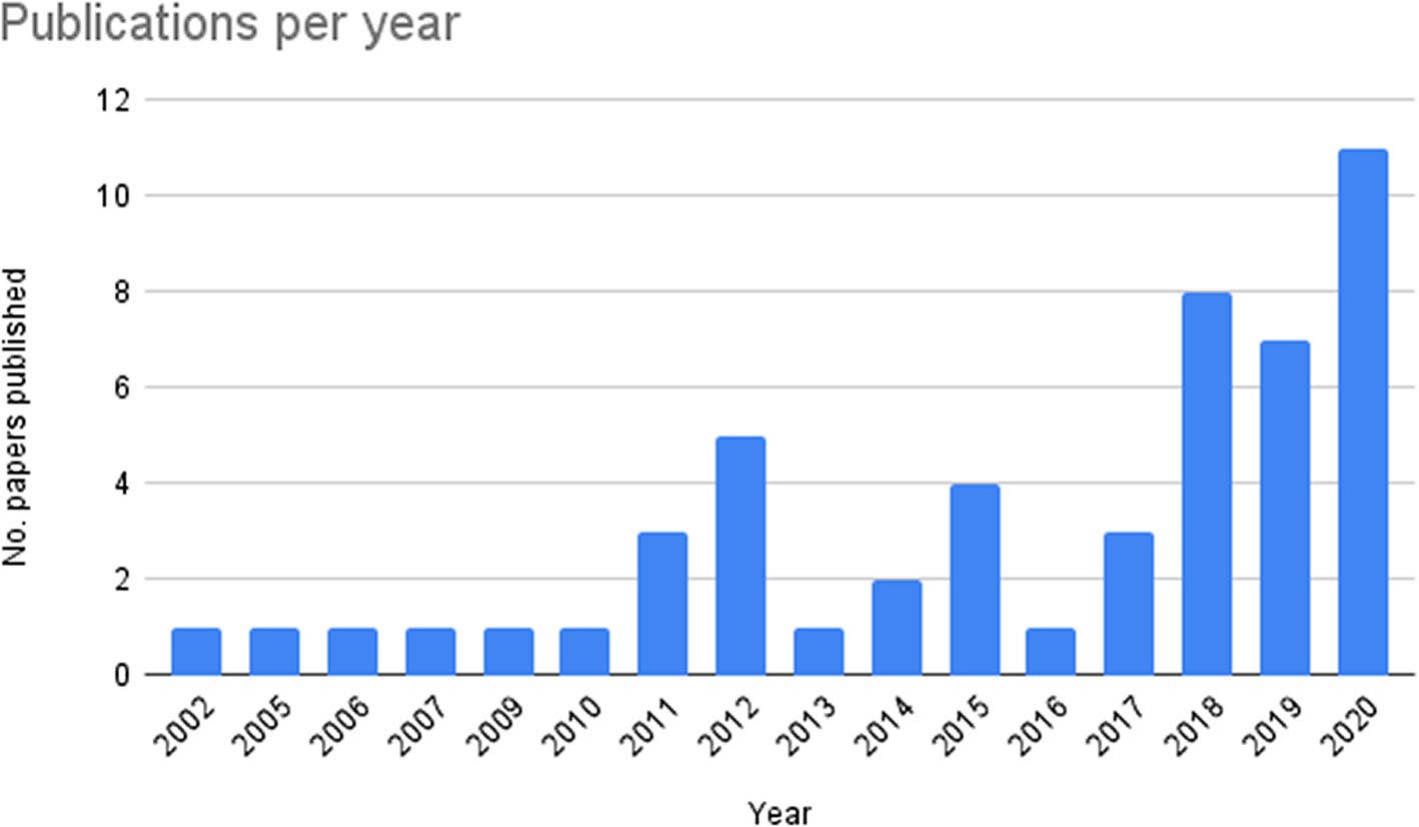
Publications per year

The results suggest that interest in agency in international students has increased over the past two decades. Although our search parameters limited the results to papers published between 2000 and 2020, the earliest paper in our sample appeared in 2002, and publication rates did not begin to increase until 2011, when three relevant papers appeared. The largest number of papers was published in 2020 (Fig. 2).

The papers in our sample included participants from various education levels. For instance, the majority of papers focused on either undergraduates (*n* = 17) or PhD students (*n* = 12), while seven papers focused on master’s students, four on general “postgraduates”, four on pre-sessional undergraduate students and six involved a mix of students from different program levels.

**Fig. 3:**
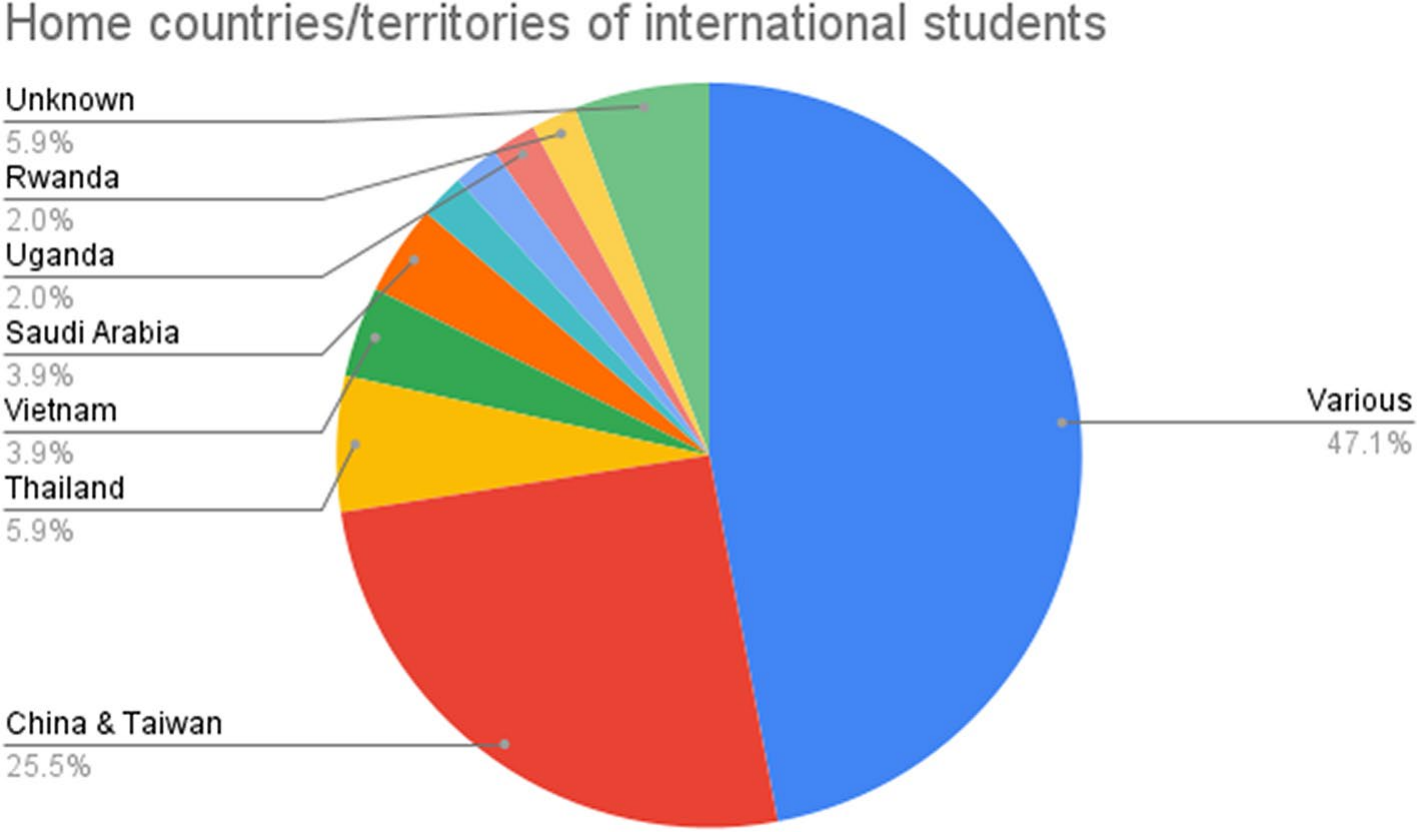
The distribution of home countries/territories

Our initial analysis also revealed trends in the populations of students studied. For instance, 13 papers (25.5%) focused on students from China and/or Taiwan. While the majority of papers included participants from various countries (*n* = 24, 47.1%), of the studies which focused on samples of participants from a single country, there was a clear emphasis on Chinese students, followed by Thailand (*n* = 3, 5.9%), Vietnam (*n* = 2, 3.9%) and Saudi Arabia (*n* = 2, 3.9%), reflecting an East and Southeast Asian focus (Fig. 3). These results suggest that Chinese international students were the most investigated group among these studies.

**Fig. 4:**
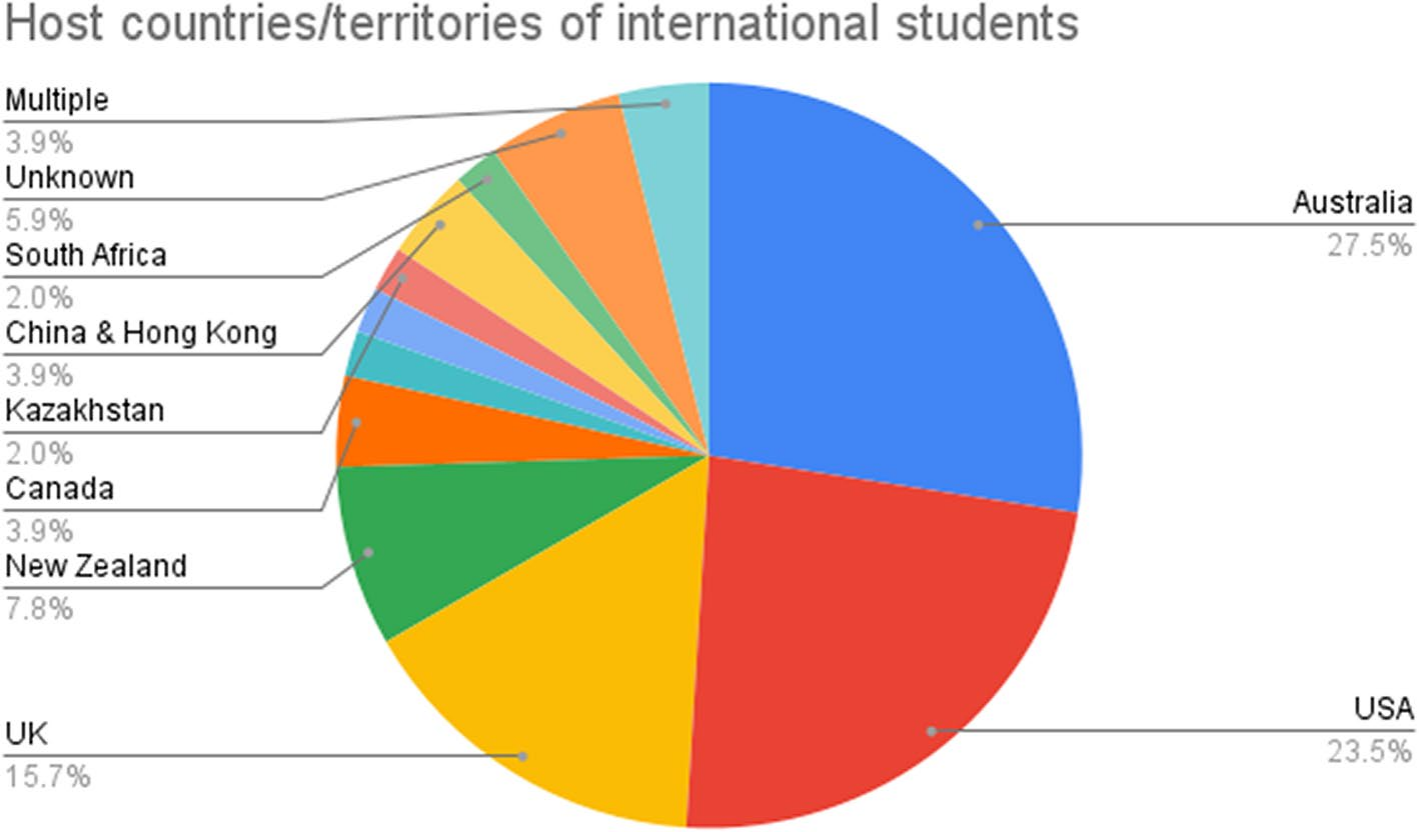
The distribution of host countries/territories

The papers in our sample focused primarily on Anglophone host countries (Fig. 4): Australia (*n* = 14; 27.5%), USA (*n* = 12; 23.5%), UK (*n* = 8; 15.7%), New Zealand (*n* = 4; 7.8%) and Canada (*n* = 2; 3.9%). This finding aligns with the comprehensive UNESCO data (2022) in that these Anglophone countries have the largest share of international students. In addition, having a few East Asian host societies (e.g. China) among the reviewed studies suggests that East Asia is also attracting researcher attention in this regard.

### Q1: How is agency positioned in the literature on student voices in international higher education?

Our analysis suggests that agency inhabits different positions in the literature based on the purpose of the study. Namely, agency has appeared in five areas: agency-as-research-object (the research focus), agency-in-conceptual/theoretical-framework, agency-emergent-in-research-findings, agency-as-given (largely undefined) and agency-in-passing (mentioned, acknowledged). See Table 3 above for definitions of these categories and the Appendix for the list of papers and coding.

#### Agency as research object

Agency-as-research-object referred to instances in which student agency was identified as the research topic or as a focus in the research question(s). In total, fifteen papers were categorised as agency-as-research-object.

In cases where agency was the research object of a study, the concept of agency was used to explore international students’ agency within a particular situation or context. Most papers (9) included in this category examined agency as exercised and developed within the educational degree programme, based on the participants’ experiences as international students. For instance, Baxter (2019) focused on the “international education space” and how Rwandan scholarship recipients “experience and exercise agency in this space” (p. 107). Similarly, Kettle (2005) investigated agency in relation to how an international student “engages with the practices of a Master of Education course” (p. 46).

Other, more specific, contexts in which international student agency has been examined included supervision settings (Chang & Strauss, 2010; Nomnian, 2017), building intercultural relationships (Kudo et al., 2020), identity development (Ingleton & Cadman, 2002) and engagement with out-of-campus contexts such as Christian churches (Yu, 2020). While intercultural relationships, identity development and church engagement were stand-alone papers, they used these situations to examine international students’ agency in adapting to their new educational or sociocultural contexts. For instance, Yu (2020) examined church participation by non-Christian Chinese international students as a way of exploring agency in cross-cultural engagement beyond the university campus. Thus, when used as a research object, agency has tended to be explored in terms of the extent of international students’ agency and how students exercise agency in negotiating new settings, cultures and practices in the host countries.

Papers examining agency-as-research-object built on a range of concepts to delineate agency in their research: Lefebvre’s (1991) threefold notion of space (Baxter, 2019); pedagogy of flow and social identities (Fotovatian, 2012); a “three-stage ecological and person-in-context conceptual framework of intercultural relationship development” (Kudo et al., 2020); needs-response agency (Nguyen & Robertson, 2020); Bandura’s theory of agency (Mukhamejanova, 2019; Nwokedi & Khanare, 2020); Pavlenko and Blackledge’s (2004) identity construction and negotiation in multilingual settings (Nomnian, 2017); agency and adaptation theory (Yu, 2020); subjectification, confidence, shame, social relationships (Ingleton & Cadman, 2002); Archer’s reflexivity (Matthews, 2017, 2018); agency in positioning theory (Tran & Vu, 2018); agency theory (Chang & Strauss, 2010); and critical discourse analysis (Kettle, 2005). Although these papers drew on varying sources in defining agency, all definitions involved the notion of acting with intention or out of personal desire, whether in a language (Fotovatian, 2012) or discursive context (Kettle, 2005), or as actors within their broader life course (Kudo et al, 2020; Matthews, 2017, 2018). In defining agency, six papers mentioned the agent’s ability to change/alter their contexts and/or recognise the interaction between agency and structure (Chang & Strauss, 2010; Fotovatian, 2012; Kettle, 2005; Nomnian, 2017; Nwokedi & Khanare, 2020; Yu, 2020).

Thus, agency is used as a research object by the literature on student experiences in IHE. According to the shared notion in the definitions of agency identified here, researchers often use agency when investigating international students’ intentional action on/interaction with structure in IHE. They were particularly interested in the varying extent and manifestation of such tendency (agency) in adapting to their new environments.

#### Agency in framework

In contrast to the previous category, agency-in-framework referred to papers in which agency was defined by or integrated into the theoretical or conceptual framework of the study but did not focus on agency as the object of inquiry. Here, a *theoretical framework* refers to cases in which researchers draw on existing theory to conceptualise the research. In contrast, a *conceptual framework* is what researchers formulate themselves for their own research purposes, because existing models cannot sufficiently explain the researched phenomena. Eighteen papers were placed into this category, with two of them double-coded with agency-as-given (Dingyloudi et al., 2019; Weng, 2020).

Out of 18 papers, 11 incorporated agency into their frameworks (see Appendix) to understand international students’ experiences. Researchers using existing definitions of agency drew on a range of theorists including Ahearn (2001); Biesta and Tedder (2007); Sen (2000); Foucault (1979); Davies (1990); Archer (2003); Bandura (2001); and Marginson (2014). Some scholars adopted not only specific definitions, but also theoretical frameworks that include/imply agency. The two most frequently used frameworks were communities of practice (CoP; Lave & Wenger, 1991) and the sociocultural definition of agency (Ahearn, 2001). CoP understands agency as a capacity required for “socialisation into specific disciplinary communities” by adjusting and appropriating the self to ultimately shape self-identity (Killick, 2013). Ahearn’s (2001) definition of agency, “socioculturally mediated capacity to act”, is quoted in several studies that identified the roles of language (Anderson, 2017); personal backgrounds and aspirations (Chang, 2011); and academic communities (Weng, 2020) in enabling or restricting student agency. It is notable that the aforementioned theories are almost exclusively rooted in Western scholarship, and have been adopted across Western and non-Western contexts. This reflects the dominant use of these perspectives in understanding agency—at least in the English literature.

Although different frameworks were used and developed to investigate various aspects of student experience in IHE, the reviewed papers seem to understand agency in fundamentally similar ways. What agency enables is students’ ability to actively negotiate social structure towards the construction of the self and environment. On the one hand, agency is a capacity to “creatively interact with” (Weber, 1964, in Amadasi & Holliday, 2018), to exploit (McAlpine, 2012), to engage with (Biesta & Tedder, 2007), to adjust to (Dai, 2020), and to improvise and even to reproduce the existing structure (Heng, 2018a, 2018b). On the other hand, agency is not only exerted on structure but also on the self (Adawu & Martin-Beltran, 2012; Kudo et al., 2019; Woo et al., 2015). For instance, Bandura’s (2001) theory of agency, used in Woo et al. (2015), focuses on self-reflexive agency that is defined as “the essence of humanness that reflects an individual’s capacity to exert control over his or her own life” (p. 290).

By including agency in theoretical and conceptual frameworks, researchers built their studies on the assumption that students are agents. This means that students are presumed to be able to engage in reciprocal interaction with their environments, through which both student and context undergoes transformation. Given that these papers provided meaningful elaborations about their research topics based on this assumption, the use of agency-in-framework offers deductively drawn evidence about the critical role of agency in adaptation and personal development of international students.

#### Agency as emergent finding

The papers in the agency-as-finding category did not include agency in their main focus or research questions, but instead discussed agency as an emergent finding. Fourteen papers fell into this category. Of these 14 papers, three discussed agency as a finding and provided a definition, while the other 11 also arrived at agency in the findings but treated the concept as a given, not providing a definition.

In the three papers that provided a definition for agency (Copland & Garton, 2011; Ding & Devine, 2018; Koehne, 2006), agency was used in the interpretation of findings from diverse perspectives. For example, Copland and Garton (2011) identified three types of agency (*self-agency*, *other-agency* and *joint-agency*) denoting to “whom agency is principally attributed” (p. 248) in international students’ daily encounters in which they had to use English for communication. Their findings empirically confirmed the sociocultural aspect of agentic practices, which are influenced by language that can enhance or reduce international students’ “capacity to act” (Ahearn, 2001, p. 251). Other findings interpreted using agency were autonomous and empowered action in supervision experiences (Ding & Devine, 2018) and students’ ability to talk about themselves and protect their self-confidence in challenging situations in IHE (Koehne, 2006).

Eleven papers in this category did not provide a specific definition of agency. These papers dealt with the agency of international students at macro, institutional and individual levels. By using a macro perspective, student agency was influenced by global geopolitical competition (Mulvey, 2020) and by the policy structures around international student security (Sawir et al., 2009). At the institutional level, international students’ agency appeared to be affected by curriculum such as liberal arts education (Bjork et al., 2020) and specific study abroad programmes (Dai et al., 2020; Dai & Garcia, 2019; deSaint-Georges et al., 2020). The remaining five papers focused on agency enacted in the individual experience, particularly individual identity development (Bond, 2019; Gonzalez & Ariza, 2015; Gu et al., 2019; Song, 2020; Wang, 2012).

The agency-as-finding category included more than a quarter of the papers in the dataset (14 out of 51). This suggests that research on student voices in IHE arrived at the concept of agency inductively. That is, even when the aim was not initially focused on agency, empirical findings pointed to agency. Agency as an emergent finding confirms the deductively assumed (by frameworks or as research objects) role of student agency in IHE.

#### Agency as given

We initially conceived of agency-as-given as a separate category during the early phases of our analysis. As explained earlier, agency-as-given means that we could not locate a definition or conceptualisation of agency in these papers. Later stages of the analysis indicated that this category is mostly a subset of other categories, with only one paper marked solely as agency-as-given (Vu & Doyle, 2014). Agency-as-finding and agency-in-passing categories account for the largest proportion of agency-as-given papers (11/14 and 3/3, respectively). Agency-in-framework had two of 19 papers and agency-as-object had no double coding of the additional agency-as-given category. These proportions make sense as the former two categories arrived at agency inductively without including the construct in their purposes, while the latter groups intentionally focused on agency either as its object or as part of their framework. In total, more than a third of the reviewed papers (17 of 51) did not discuss what agency is or how it is conceptualised. This suggests an implicit assumption that the meaning of agency is apparent and universal, which may not be true.

#### Agency in passing

This category includes papers in which agency briefly appears and is not the focus of the study. In these papers, agency is mentioned only a few times; hence, they appeared in our search results. However, they neither elaborate on the concept nor provide a definition for it. Simply, the authors in these papers were not interested in agency as their main concern, but acknowledged the presence of agency in international student lives.

The systematic search yielded three entries that fell into this category (Clerehan et al., 2012; Mayuzumi et al., 2007; Wang, 2018). In these studies, agency was touched upon briefly when discussing how international students felt empowered and “saw themselves as future change agents” (Clerehan et al., 2012, p. 215) or when reflexive thinking for intercultural experiences (Mayuzumi et al., 2007) and problems encountered in academic development and personal growth engage in reflexive thinking (Wang, 2018).

This category strengthens our argument regarding the buzzword treatment of agency in IHE research. The brief appearance of agency signals the relevant but insignificant position of agency, spreading the concept to a range of areas in IHE but on a superficial level.

### Q2: How can the literature on international student agency be conceptually synthesised?

The findings suggest that international student agency has been positioned as a research object, as part of the study framework, as a research finding, as a given and as a passing construct. Agency has been either explicitly defined or implicitly explained to varying degrees of clarity. Agency tends to be most directly and clearly defined as a research object or in frameworks, while least so in papers coded agency-in-passing. We explored the various definitions of agency to develop an integrative understanding of international students’ agency, as described by students themselves and as shared in the literature.

We inductively developed a framework that incorporates the overlapping or contrasting conceptions of agency. While researchers seem to implicitly agree on a similar *construct* of agency, the contents (*functions*, *mediators* and *outcomes* of agency) appear differently.

#### Construct of agency

When researchers define agency in their studies, they often conceptualise it in relation to structure. This is because most of the original theories of agency to which these studies referred originated in sociology. Thus, to develop a comprehensive understanding of agency, it is necessary to clarify how agency interacts with structure in various situations. According to this review, international students’ agency is expressed in how students actively negotiate social structures such as general learning environments (Cotterall, 2015), living contexts (Marginson, 2014), specific programmes in IHE (Baxter, 2019; Fotovatian, 2012; Kettle, 2005), supervision meetings (Chang & Strauss, 2010; Ding & Devine, 2018; Nomnian, 2017; Woo et al., 2015), intercultural relationships (Kudo et al., 2020) and disciplinary or academic communities (Anderson, 2017; Dingyloudi et al., 2019; Elliot et al., 2016; Weng, 2020).

The literature suggests that between structure and agency exists a mediated interaction involving various sociocultural and conditioning factors. Ahearn’s (2001) definition of agency as a capacity that is mediated by social and cultural factors was frequently cited in papers that attempted to use agency to explain a range of phenomena in IHE (Anderson, 2017; Chang, 2011; Weng, 2020). Studies have also shown the role of other social subjects in conditioning students’ agency practice, which supports Ahearn’s claim that agency cannot be conceptualised without considering various mediators. For instance, Copland and Garton (2011) found that agency is exercised by the self, others or even jointly, which also provides empirical supports for Bandura’s (2001) agency framework (as in Woo et al., 2015).

**Fig. 5:**
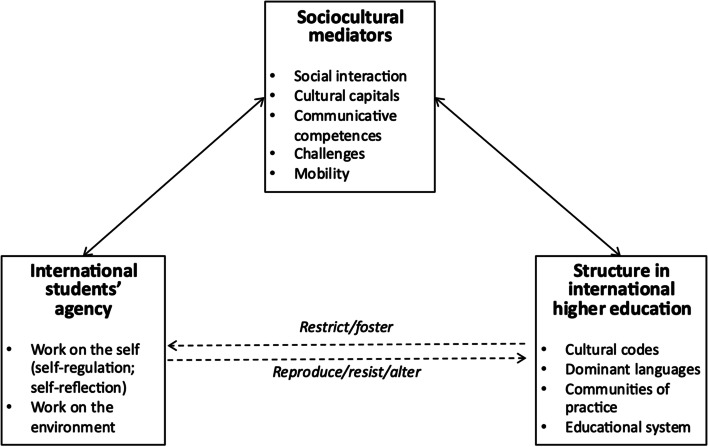
A general construct of agency in the literature on international students in higher education

As illustrated in Fig. 5, a general construct of student agency, incorporating the inseparable structure and sociocultural mediators, emerged from the current review. The figure will be discussed in more detail in the following subsections.

#### Functions of agency

Researchers have interpreted students’ various actions and statuses as agentic, leading to challenges in distinguishing what constitutes agency and what does not. To prevent the term “agency” from becoming a buzzword, there is a need to integrate the previous findings and identify shared features of agency. Overall, student agency seems to be manifested in actions that are taken internally and externally, both on the self and environment.

The first function of agency is self-reflection, or intrapersonal deliberation about the self, such as engaging in “self-talk” to fight feelings of demoralisation (Koehne, 2006). This is similar to Archer’s (2003) theory in which internal conversation is used as a tool for exercising agency (in Matthews, 2017, 2018; Yu, 2020). Self-reflection involves knowing the desires of the self, which is necessary for students to intentionally take actions (Fotovatian, 2012; Kettle, 2005) as actors of their own becoming within their life course (Kudo et al, 2020; Tran & Vu, 2018; Marginson, 2014; Matthews, 2017, 2018).

The second function of agency is behavioural self-regulation, including students’ adjustment and appropriation of the self in the host countries (Heng, 2018a, 2018b; Mukhamejanova, 2019; Wang, 2018; Yu, 2020). This involves developing and applying strategies for successful functioning in IHE. Such work on the self is triggered by contextual factors. For instance, the frequently identified or assumed manifestation of agency is to engage with, negotiate, or develop strategies to overcome the challenges that accompany studying abroad (Heng, 2018a, 2018b; Woo et al., 2015).

This is linked to the other function of agency: working on the environment. In the literature, agency was conceptualised to allow students to resist (Tran & Vu, 2018), or accept and exploit the environment (Cotterall, 2015) and change/alter their environment (Chang & Strauss., 2010; Fotovatian, 2012; Kettle, 2005; Nomnian, 2017; Nwokedi & Khanare, 2020; Yu, 2020). Students’ internal conversation with the self, and external expression of agency on the self and on the environment, suggests that student agents are empowered, autonomous subjects in IHE, who have the locus of control. In short, the commonly acknowledged core function of agency is to enable students to intentionally act on their own thoughts and actions, by reflecting on and regulating the surrounding environment.

#### Mediators of agency

The functions of international students’ agency are mediated by sociocultural factors as shown in Fig. 5. The mediating impacts are attributed to what the students bring and what the new context in the host countries can offer. Previous research suggests that international students’ agency is fostered or restricted by cultural capitals (Cotterall, 2015; Weng, 2020), personal values and aspirations (Chang, 2011) and communicative competences (Anderson, 2017; Copland & Garton, 2011; Sawir et al., 2012; Weng, 2020). These resources are employed by international students when they apply their agency in relation to the new environments in the host countries, such as unfamiliar communities with different affordances (Kudo et al., 2019), which require different roles and trigger different reactions.

Furthermore, mobility, which entails immersion into a novel context, produces additional challenges for international students. While a focus on adaptation difficulties could reproduce deficit models of IHE, studies in this paper highlighted students’ use of agency in coping with challenges. From this perspective, interactions with new academic, cultural and social settings in IHE are not a barrier for success but a catalyst for promoting agency. This might explain why certain moments of interaction (e.g. supervision meetings, intercultural communication) and tools for interaction (e.g. language, communicative competences) in IHE were frequently studied contexts aimed at producing data about student agency.

#### Outcomes of agency

The final section synthesises construct, functions and mediators of agency to elaborate on the outcomes of enacting agency in IHE. By using self-reflexive and self-regulative functions of agency, international students achieve personal growth and identity development (Adawu & Martin-Beltran, 2012; Cotterall, 2015; Killick, 2013). Concepts like self-formation (Marginson, 2014) and agency-for-becoming (Tran & Vu, 2018) are examples of how agency may influence augmentation of the self in IHE.

Agency also leads to successful socialisation and transition into the new communities. As academic and sociocultural adaptation requires agency in transforming the self (Anderson, 2017), this outcome follows the first outcome of agency, self-formation. However, by locating agency within the boundary of structure, socialisation results in reproducing the existing structure. A more transformative outcome is when student agents construct their own environments, not only adjusting to the given one. This includes creating a more global learning environment by intentionally pursuing IHE (Chang, 2011). Thus, international students’ agency may result in formation of the self and reproductive and productive formation of the environment.

## Conclusions and recommendations

International higher education has become an important topic of policy and scholarly discussion in the last two decades, and international students have attracted the greatest attention in the growing literature (Gümüş et al., 2020). Yet, international student agency has been mostly neglected (Volet & Jones, 2012) and only recently began to attract more attention. Thus, this study systematically reviewed the existing scholarly studies on IHE that specifically included student agency in their abstracts, titles or keywords and focused on international student voices rather than professors or lecturers. As a response to the identified issue of agency as a buzzword, an integrated conceptualisation of international student agency was constructed in this study. Theoretically, we established the interrelationship between different approaches to international students’ agency and brought them into a structure, through which existing and future research can build on each other. Methodologically, we formulated a helpful tool for collecting and analysing empirical data, which can guide researchers what to focus on or what are the manifestations of international students’ agency.

Reviewed papers mostly focused on mobility to the West, particularly Chinese students in Anglo-American countries. Participants in 17 out of 51 studies (33.33%) were all or mostly Chinese students, while only six of the total papers (11.77%) collected data from students in non-Anglo-American countries (e.g. China, South Africa, Kazakhstan and Japan). This can be problematic because it is not the direction of mobility that determines students’ agency; international students studying in less popular destinations also possess and practice agency. As this gap in the literature was also pointed out by previous researchers (e.g. Lipura & Collins, 2020; Waters & Brooks, 2013) in their recent review on international student mobility, it reflects the general trend in research on IHE. Two neglected populations of international students emerge: non-Chinese students and students in less recognised study abroad destinations. This pattern reflects the current mobility trends in IHE and researchers’ endeavours to reshape the historically entrenched deficit narratives around Chinese students. Future research might address this gap by examining agency of these two under-researched groups of students.

Furthermore, a methodological limitation of the reviewed papers is a paucity of longitudinal studies. Only eight studies among the reviewed traced students’ agency throughout multiple data collection points. This indicates the static conceptualisation of agency, which limits capturing changes in agency and structure through higher education. If IHE is to be understood as a process of students’ agentic self-formation (Marginson, 2014), how mobile students develop their agency during their higher education experiences and what the outcomes of their agency development are should be clarified. This can be done by providing more longitudinal perspectives on international students’ agency.

Another important limitation of the reviewed papers is the lack of consistency in conceptualising and defining agency. A third of the selected papers fell into the agency-as-given category; that is, we could not locate any definition or conceptualisation in these papers, the meaning is assumed. We initially thought agency-as-given category as a separate one from the others during the early analysis of the systematically selected papers. The later stages of the analysis indicated that this category is a subset to existing other categories. In total, 17 out of 51 papers (~ 33%) did not discuss what agency is or how it is conceptualised although they included it in their studies. This could be because, firstly, researchers may not know what it means or find it challenging to provide an explicit definition of agency; or because of an implicit assumption that agency is so commonly discussed that everyone understands the same meaning from it, which is not true as the next paragraph explains.

The papers that specified what agency is adopted a range of different frameworks, implying a lack of shared understanding of agency and that researchers rarely build upon each other’s work. This might be a necessary phase for an embryonic field that has to experience unintegrated and multiple, separate drivers until a few emerge as dominant and more acknowledged frameworks. The present review, therefore, contributes a significant step towards the next phase of the field by synthesising the burgeoning discussions about international students’ agency.

## Supplementary Information

Below is the link to the electronic supplementary material.

Supplementary file1 (DOCX 55 KB)

## Footnotes

1 We appreciate Simon Marginson for alerting us to this point.
