# Supplementary material for: A systematic review of student agency in international higher education
Source: High Educ (Dordr). 2022 Nov 19:1–21. Online ahead of print. doi: 10.1007/s10734-022-00952-3 (PMC9676751; doi:10.1007/s10734-022-00952-3)
Supplement: Supplementary file 1 — Supplementary file1 (DOCX 55 KB) [file 10734_2022_952_MOESM1_ESM.docx]

# Appendix

| Paper citation | Coding category | Method | Sending Country/  region | Host Country/  region |
| --- | --- | --- | --- | --- |
| Adawu, A., & Martin-Beltran, M. (2012). Points of Transition: Understanding the Constructed Identities of L2 Learners/Users across Time and Space. *Critical Inquiry in Language Studies*, *9*(4), 376–400. https://doi.org/http://dx.doi.org/10.1080/15427587.2012.664038 | agency-in-framework | QL | China, India, Sri Lanka | US |
| Amadasi, S., & Holliday, A. (2018). “I Already Have a Culture.” Negotiating Competing Grand and Personal Narratives in Interview Conversations with New Study Abroad Arrivals. *Language and Intercultural Communication*, *18*(2), 241–256. https://doi.org/http://dx.doi.org/10.1080/14708477.2017.1357727 | agency-in-framework | QL | unclear | UK |
| Anderson, T. (2017). The doctoral gaze: Foreign PhD students’ internal and external academic discourse socialization. *Linguistics and Education*, *37*, 1–10. https://doi.org/http://dx.doi.org/10.1016/j.linged.2016.12.001 | agency-in-framework | QL | China | Canada |
| Baxter, A. (2019). Engaging Underrepresented International Students as Partners: Agency and Constraints Among Rwandan Students in the United States. *Journal of Studies in International Education*, *23*(1), 106–122. https://doi.org/10.1177/1028315318810858 | agency-as-research-object | QL | Rwanda | US |
| Bjork, C., Abrams, A., Hutchinson, L. S., & Kyrkjebo, N. I. (2020). “Don’t Change Yourselves”: International Students’ Concepts of Belonging at a Liberal Arts College. *Journal of International Students*, *10*(3), 553–570. https://doi.org/10.32674/jis.v10i3.969 | agency-as-finding; agency-as-given | QL | Mixed | US |
| Bond, B. (2019). International Students: Language, Culture and the “Performance of Identity.” *Teaching in Higher Education*, *24*(5), 649–665. https://doi.org/http://dx.doi.org/10.1080/13562517.2019.1593129 | agency-as-finding; agency-as-given | QL | Mixed (mostly Chinese) | UK |
| Chang, C. E., & Strauss, P. (2010). “Active agents of change?” Mandarin-speaking students in New Zealand and the thesis writing process. *Language and Education*, *24*(5), 415–429. https://doi.org/http://dx.doi.org/10.1080/09500781003789873 | agency-as-research-object | MX | China, Taiwan | New Zealand |
| Chang, Y.-J. (2011). Picking One’s Battles: NNES Doctoral Students’ Imagined Communities and Selections of Investment. *Journal of Language, Identity, and Education*, *10*(4), 213–230. https://doi.org/http://dx.doi.org/10.1080/15348458.2011.598125 | agency-in-framework | QL | Taiwan | US |
| Clerehan, R., Mccall, L., Mckenna, L., & Alshahrani, K. (2012). Saudi Arabian nurses’ experiences of studying Masters degrees in Australia. *International Nursing Review*, *59*(2), 215–221. https://doi.org/10.1111/j.1466-7657.2011.00951.x | agency-in-passing; agency-as-given | QL | Saudi Arabian | Australia |
| Copland, F., & Garton, S. (2011). “I felt that i do live in the UK now”: International students’ self-reports of their english language speaking experiences on a pre-sessional programme. *Language and Education*, *25*(3), 241–255. https://doi.org/10.1080/09500782.2011.566617 | agency-as-finding | QL | Mixed (mostly Chinese) | UK |
| Cotterall, S. (2015). The rich get richer: International doctoral candidates and scholarly identity. *Innovations in Education and Teaching International*, *52*(4), 360–370. https://doi.org/http://dx.doi.org/10.1080/14703297.2013.839124 | agency-in-framework | QL | China, India, Indonesia, Mongolia, North America, Kenya | Australia |
| Dai, K. (2020). Learning between two systems: a Chinese student’s reflexive narrative in a China-Australia articulation programme. *Compare*, *50*(3), 371–390. https://doi.org/10.1080/03057925.2018.1515008 | agency-in-framework | QL | China | Australia |
| Dai, K., & Garcia, J. (2019). Intercultural learning in transnational articulation programs: The hidden agenda of Chinese students’ experiences. *Journal of International Students*, *9*(2), 362–383. https://doi.org/10.32674/jis.v9i2.677 | agency-as-finding; agency-as-given | QL | China | Australia |
| Dai, K., Matthews, K. E., & Reyes, V. (2020). Chinese Students’ Assessment and Learning Experiences in a Transnational Higher Education Programme. *Assessment & Evaluation in Higher Education*, *45*(1), 70–81. https://doi.org/http://dx.doi.org/10.1080/02602938.2019.1608907 | agency-as-finding; agency-as-given | QL | China | Australia |
| De Saint-Georges, I., Budach, G., & Tress, C. (2020). “We need to become ‘educational chameleons’”: from unified to multiple norms in a multilingual and international higher education context. *European Journal of Applied Linguistics*, *8*(2), 233–256. https://doi.org/10.1515/eujal-2020-0011 | agency-as-finding; agency-as-given | QL | Mixed | Luxembourg |
| Ding, Q., & Devine, N. (2018). Exploring the supervision experiences of Chinese overseas PhD students in New Zealand. *Knowledge Cultures*, *6*(1), 62–78. https://doi.org/http://dx.doi.org/10.22381/KC6120186 | agency-as-finding | QL | China | New Zealand |
| Dingyloudi, F., Strijbos, J.-W., & de Laat, M. F. (2019). Value creation: What matters most in Communities of Learning Practice in higher education. *Studies in Educational Evaluation*, *62*, 209–223. https://doi.org/10.1016/j.stueduc.2019.05.006 | agency-in-framework; agency-as-given | QL | Mixed | Germany |
| Elliot, D. L., Baumfield, V., Reid, K., & Makara, K. A. (2016). Hidden treasure: successful international doctoral students who found and harnessed the hidden curriculum. *Oxford Review of Education*, *42*(6), 733–748. https://doi.org/10.1080/03054985.2016.1229664 | agency-in-framework | QL | Mixed | UK |
| Fotovatian, S. (2012). Three constructs of institutional identity among international doctoral students in Australia. *Teaching in Higher Education*, *17*(5), 577–588. https://doi.org/http://dx.doi.org/10.1080/13562517.2012.658557 | agency-as-research-object | QL | China, Indonesia, Nepal | Australia |
| González, J. J. V., & Ariza, J. A. A. (2015). From Awareness to Cultural Agency: EFL Colombian Student Teachers’ Travelling Abroad Experiences. *Profile*, *17*(1), 123–141. https://doi.org/http://dx.doi.org/10.15446/profile.v17n1.39499 | agency-as-finding; agency-as-given | MX | Colombia | US |
| Gu, M. M., Guo, X. G., & Lee, J. C. K. (2019). The interplay between ethnic and academic identity construction among South Asian students in Hong Kong tertiary education. *Higher Education*, *78*(6), 1109–1127. https://doi.org/10.1007/s10734-019-00391-7 | agency-as-finding; agency-as-given | QL | India, Philippines, Pakistan | Hong Kong |
| Heng, T. T. (2018a). Coping strategies of international Chinese undergraduates in response to academic challenges in U.S. colleges. *Teachers College Record*, *120*(2). https://www.scopus.com/inward/record.uri?eid=2-s2.0-85044141380&partnerID=40&md5=41674c8cc8e273f544d7da68cebd3352 | agency-in-framework | QL | China | US |
| Heng, T. T. (2018b). Different is not deficient: contradicting stereotypes of Chinese international students in US higher education. *Studies in Higher Education*, *43*(1), 22–36. https://doi.org/10.1080/03075079.2016.1152466 | agency-in-framework | QL | China | US |
| Heng, T. T. (2018c). Chinese International Students’ Advice to Incoming First-Year Students: Involving Students in Conversations With Them, Not About Them. *Journal of College Student Development*, *59*(2), 232–238. https://doi.org/http://dx.doi.org/10.1353/csd.2018.0020 | agency-in-framework | QL | China | US |
| Ingleton, C., & Cadman, K. (2002). Silent issues for international postgraduate research students: Emotion and agency in academic success. *Australian Educational Researcher*, *29*(1), 93–113. https://doi.org/http://dx.doi.org/10.1007/BF03219771 | agency-as-research-object | QL | Egypt, India, Indonesia, Peru and Thailand | Australia |
| Kettle, M. (2011). Academic practice as explanatory framework: Reconceptualising international student academic engagement and university teaching. *Discourse*, *32*(1), 1–14. https://doi.org/10.1080/01596306.2011.537067 | agency-in-framework | QL | Thailand | Australia |
| Kettle, M. (2005). Agency as Discursive Practice: From “nobody” to “somebody” as an international student in Australia1. *Asia Pacific Journal of Education*, *25*(1), 45–60. https://doi.org/10.1080/02188790500032525 | agency-as-research-object | QL | Thailand | Australia |
| Killick, D. (2013). Global citizenship, sojourning students and campus communities. *Special Issue: Making Sense of Teaching in Difficult Times.*, *18*(7), 721–735. https://doi.org/http://dx.doi.org/10.1080/13562517.2013.836087 | agency-in-framework | QL | Mixed | Europe and Australia |
| Koehne, N. (2006). (Be)coming, (Be)longing: Ways in which international students talk about themselves. *Discourse*, *27*(2), 241–257. https://doi.org/10.1080/01596300600676219 | agency-as-finding | QL | Mixed | Australia |
| Kudo, K., Volet, S., & Whitsed, C. (2020). Intercultural relationship development and higher education internationalisation: a qualitative investigation based on a three-stage ecological and person-in-context conceptual framework. *Higher Education*, *80*(5), 913–932. https://doi.org/10.1007/s10734-020-00523-4 | agency-as-research-object | QL | Mixed | Japan |
| Kudo, K., Volet, S., & Whitsed, C. (2019). Development of intercultural relationships at university: a three-stage ecological and person-in-context conceptual framework. *Higher Education*, *77*(3), 473–489. https://doi.org/10.1007/s10734-018-0283-9 | agency-in-framework | QL | Mixed | Japan |
| Marginson, S. (2014). Student self-formation in international education. *Journal of Studies in International Education*, *18*(1), 6–22. https://doi.org/10.1177/1028315313513036 | agency-in-framework | QL | Mixed | Australia, New Zealand |
| Matthews, B. (2017). “I wouldn’t imagine having to go through all this and still be the same person. No way”: structure, reflexivity and international students. *Journal of Research in International Education*, *16*(3), 265–278. https://doi.org/10.1177/1475240917745611 | agency-as-research-object | MX | Mixed | UK |
| Matthews, B. (2018). “You just use your imagination and try to fix It”: Agential change and international students. *Journal of International Students*, *8*(1), 332–350. https://doi.org/10.5281/zenodo.1134311 | agency-as-research-object | QL | Russia, Libya | UK |
| Mayuzumi, K., Motobayashi, K., Nagayama, C., & Takeuchi, M. (2007). Transforming Diversity in Canadian Higher Education: A Dialogue of Japanese Women Graduate Students. *Teaching in Higher Education*, *12*(5–6), 581–592. https://doi.org/https://doi.org/10.1080/13562510701595200 | agency-in-passing; agency-as-given | QL | Japan | Canada |
| Mukhamejanova, D. (2019). International students in Kazakhstan. *International Journal of Comparative Education and Development*, *21*(3), 146–163. https://doi.org/10.1108/IJCED-07-2018-0024 | agency-as-research-object | QL | Mixed | Kazakhstan |
| Mulvey, B. (2020). International Higher Education and Public Diplomacy: A Case Study of Ugandan Graduates from Chinese Universities. *Higher Education Policy*, *33*(3), 459–477. https://doi.org/10.1057/s41307-019-00174-w | Agency-as-finding; agency-as-given | QL | Uganda | China |
| Nguyen, M., Robertson, M. J., Nguyet Nguyen, M., & Robertson, M. J. (2020). International students enacting agency in their PhD journey. *Teaching in Higher Education*, *0*(0), 1–17. https://doi.org/10.1080/13562517.2020.1747423 | agency-as-research-object | QL | Vietnam | Australia |
| Nomnian, S. (2017). Thai PhD Students and Their Supervisors at an Australian University: Working Relationship, Communication, and Agency. *PASAA: Journal of Language Teaching and Learning in Thailand*, *53*, 26–58. https://search.proquest.com/scholarly-journals/thai-phd-students-their-supervisors-at-australian/docview/1969012754/se-2?accountid=13042 | agency-as-research-object | QL | Thailand | Australia |
| Nwokedi, P. G., & Khanare, F. P. M. K. (2020). Thriving in the Face of Adversity: Mapping Experiences of International Students in a South African Higher Education Institution. *Journal of Comparative and International Higher Education*, *12*(1), 49–66. https://doi.org/https://doi.org/10.32674/jcihe.v12iSpring.1400 | agency-as-research-object | QL | Mixed | South Africa |
| Sawir, E., Marginson, S., Nyland, C., Ramia, G., & Rawlings-Sanaei, F. (2009). The pastoral care of international students in New Zealand: Is it more than a consumer protection regime? *Asia Pacific Journal of Education*, *29*(1), 45–59. https://doi.org/10.1080/02188790802655049 | agency-as-finding; agency-as-given | QL | Mixed | New Zealand |
| Sawir, E., Marginson, S., Forbes-Mewett, H., Nyland, C., & Ramia, G. (2012). International Student Security and English Language Proficiency. *Journal of Studies in International Education*, *16*(5), 434–454. https://doi.org/http://dx.doi.org/10.1177/1028315311435418 | agency-as-research-object | QL | Mixed Asian Countries | Australia |
| Song, J. (2020). Contesting and Negotiating Othering from Within: A Saudi Arabian Female Student’s Gendered Experiences in the U.S. *Journal of Language, Identity, and Education*, *19*(3), 149–162. https://doi.org/http://dx.doi.org/10.1080/15348458.2019.1654386 | agency-as-finding; agency-as-given | QL | Saudi Arabia | US |
| Soong, H., Tran, L. T., & Hiep, P. H. (2015). Being and becoming an intercultural doctoral student: reflective autobiographical narratives. *Reflective Practice*, *16*(4), 435–448. https://doi.org/http://dx.doi.org/10.1080/14623943.2015.1077633 | agency-in-framework | QL | Singapore, Vietnam | Australia |
| Tran, L. T., & Vu, T. T. P. (2018). ‘Agency in mobility’: towards a conceptualisation of international student agency in transnational mobility. *Educational Review*, *70*(2), 167–187. https://doi.org/10.1080/00131911.2017.1293615 | agency-as-research-object | QL | Mixed | Australia |
| Vu, H., & Doyle, S. (2014). Across borders and across cultures: Vietnamese students’ positioning of teachers in a university twinning programme. *Journal of Education for Teaching*, *40*(3), 267–283. https://doi.org/10.1080/02607476.2014.903026 | agency-as-given | QL | Vietnam | New Zealand |
| Wang, I. K.-H. (2018). Long-Term Chinese Students’ Transitional Experiences in UK Higher Education: A Particular Focus on Their Academic Adjustment. *International Journal of Teaching and Learning in Higher Education*, *30*(1), 12–25. https://search.proquest.com/scholarly-journals/long-term-chinese-students-transitional/docview/2024001663/se-2?accountid=13042 | agency-in-passing; agency-as-given | MX | China | UK |
| Wang, Y. (2012). Transformations of Chinese international students understood through a sense of wholeness. *Teaching in Higher Education*, *17*(4), 359–370. https://doi.org/10.1080/13562517.2011.641004 | agency-as-finding; agency-as-given | QL | China | Canada |
| Weng, T. (2020). On becoming a doctoral student: Chinese doctoral students’ socialization of capital and habitus in academia. *British Journal of Sociology of Education*, *41*(4), 555–573. https://doi.org/http://dx.doi.org/10.1080/01425692.2020.1745056 | agency-in-framework; agency-as-given | QL | China | US |
| Woo, H., Jang, Y. J., &Henfield, M. S. (2015). International Doctoral Students in Counselor Education: Coping Strategies in Supervision Training. *Journal of Multicultural Counseling and Development*, *43*(4), 288–304. https://doi.org/http://dx.doi.org/10.1002/jmcd.12022 | agency-in-framework | QL | Mixed | US |
| Yu, Y. (2020). From universities to Christian churches: agency in the intercultural engagement of non-Christian Chinese students in the UK. *Higher Education*, *80*(2), 197–213. https://doi.org/10.1007/s10734-019-00474-5 | agency-as-research-object | MX | China | UK |
